# Supplementary material for: Clinical Characteristics of Acute Hepatitis E and Their Correlation with HEV Genotype 3 Subtypes in Italy
Source: Pathogens. 2020 Oct 11;9(10):832. doi: 10.3390/pathogens9100832 (PMC7650787; doi:10.3390/pathogens9100832)
Supplement: Supplementary file 1 [file pathogens-09-00832-s001.zip › Minosse et al supplementary data/Minosse et al_Table S1.docx]

**Table S1**. Baseline demographic characteristics and underlying comorbidities in acute HEV GT3-infected patients by severity of acute hepatitis E.

| **Characteristics** | **Non severe acute hepatitis**  **(n=24)** | **Severe acute hepatitis**  **(n=13)** | **P** |
| --- | --- | --- | --- |
| Age, years ( mean ±SD) | 60 ± 10 | 60 ± 14 | 0.99 |
| Male sex (n, %) | 19 (79.2%) | 13 (100%) | 0.3 |
| BMI, Kg/m^2^ | 24.1 ± 2.7 | 26.1 ± 3.8 | 0.20 |
| Alcohol user, (n, %) | 8/19 (42.1%) | 3/9 (33.3%) | 0.70 |
| Smoker, (n, %) | 5/19 (26.3%) | 3/7 (42.8%) | 0.63 |
| PWID, (n, %) | 0/24 (0%) | 2/11 (18.2%) | 0.2 |
| Chronic liver disease, (n, %) | 0/24 (0%) | 3/13 (23.1%) | 0.07 |
| Number of comorbidities  0  1  2  3  4  5 | 8 (33.3%)  8 (33.3%)  1 (4.2%)  4 (16.7%)  1 (4.2%)  2 (8.3%) | 2 (15.4%)  7 (53.8%)  1 (7.7%)  3 (23.1%)  0 (0%)  0 (0%) | 0.44  0.14  1  0.68 |
| Diabetes, (n, %) | 8 (33.3%) | 5 (38.5%) | 1 |
| Cardiovascular diseases, (n, %) | 5 (20.8%) | 3 (23.1%) | 1 |
| Lipid disorders, (n, %) | 5 (20.8%) | 2 (15.4%) | 1 |
| Renal diseases, (n, %) | 1 (4.2%) | 0 (0%) | 0.7 |
| Arterial hypertension, (n, %) | 10 (41.7%) | 7 (53.8%) | 0.48 |
| Digestive diseases, (n, %) | 2 (8.3%) | 1 (7.7%) | 1 |
| Respiratory diseases, (n, %) | 3 (12.5%) | 0 (0%) | 0.6 |
| Neoplastic diseases, (n, %) | 2 (8.3%) | 0 (0%) | 0.9 |
