# Supplementary material for: Clinical Characteristics of Acute Hepatitis E and Their Correlation with HEV Genotype 3 Subtypes in Italy
Source: Pathogens. 2020 Oct 11;9(10):832. doi: 10.3390/pathogens9100832 (PMC7650787; doi:10.3390/pathogens9100832)
Supplement: Supplementary file 1 [file pathogens-09-00832-s001.zip › Minosse et al supplementary data/Minosse et al_Table S2.docx]

**Table S2.** Clinical characteristics and laboratory results for acute HEV GT3-infected patients by severity of acute hepatitis E.

| **Characteristics** | **Non severe acute hepatitis**  **(n=24)** | **Severe acute hepatitis**  **(n=13)** | **P** |
| --- | --- | --- | --- |
| Duration of hospitalization, days | 8 (6.2-9) | 11 (9.5-21.5) | 0.001 |
| Duration of symptoms, days | 7 (5-9.7) | 5 (2-9) | 0.3 |
| Symptoms  Asthenia  Fever  Nausea/Vomiting  Diarrhea  Epigastric pain  Arthralgia | 12 (50%)  10 (41.7%)  10 (41.7%)  2 (8.3%)  7 (29.2%)  4 (16.7%) | 6 (46.1%)  2 (15.4%)  5 (38.5%)  2 (15.4%)  6 (46.1%)  0 (0%) | 1  0.1  1  0.6  0.5  0.4 |
| Extrahepatic manifestations | 6 (25%) | 5 (38.5%) | 0.5 |
| Laboratory parameters at peak |  |  |  |
| AST, U/L | 1008.5 (450.2-1404.5) | 2050 (303-2608.5) | 0.3 |
| ALT, U/L | 1671 (1067.5-2676) | 2167 (800-3161) | 0.7 |
| Total bilirubin, mg/dL | 3.6 (1.3-7.4) | 15.2 (11-26.5) | <0.001 |
| γGT, U/L | 300 (195.2-421.7) | 263 (107-393.5) | 0.2 |
| ALP | 318 (205-632) | 259 (189-493.5) | 0.5 |
| INR | 1.14 (1.06-1.2) | 1.5 (1.2-2.1) | 0.002 |
